# Validity and reliability of a computerized cognitive function evaluation battery (CogEvo) as a screening tool

**DOI:** 10.1002/pcn5.67

**Published:** 2023-01-06

**Authors:** Yoichi Sawada, Toru Satoh, Hideaki Saba, Hiroshi Kitamoto, Yoshiki Kato, Yoshiko Shiozuka, Tomoko Kuwada, Kana Murakami, Megumi Sasaki, Yudai Abe, Kaori Harano

**Affiliations:** ^1^ Department of Contemporary Welfare, Faculty of Health and Welfare Okayama Prefectural University Soja Okayama Japan; ^2^ Department of Neurological Surgery Ryofukai Satoh Neurosurgical Hospital Fukuyama Hiroshima Japan; ^3^ Department of Rehabilitation Ryofukai Satoh Neurosurgical Hospital Fukuyama Hiroshima Japan; ^4^ Department of Human Welfare, Faculty of Human Relations Otsuma Women's University Chiyoda‐ku Tokyo Japan

**Keywords:** CogEvo, cognitive decline, computerized cognitive function evaluation battery, dementia, validity and reliability

## Abstract

**Aim:**

The aim of this study was to determine the validity and reliability of cognitive function evaluation battery, CogEvo, a recently developed computerized cognitive function evaluation battery, as a screening tool for decreased cognitive function.

**Methods:**

The study sample comprised 123 (age: 57–97 years) community‐dwelling elderly people. They were required to perform five CogEvo tasks and complete two questions‐based neuropsychological tests, including the Mini‐Mental State Examination, so that the correlations could be analyzed. The validity and reliability of CogEvo were examined using factor analysis, MacDonald's omega reliability coefficient, logistic regression analysis, and receiver operating characteristic curve analysis.

**Results:**

Exploratory factor analysis revealed the orientation/spatial cognitive function (orientation and spatial cognition) and attention/executive function (attention, memory, and execution) factors. Structural validity was supported by confirmatory factor analysis. All two‐factor‐based subtasks showed adequate internal consistency (MacDonald's omega ≥0.6). The total CogEvo score and two‐factor scores were significantly correlated with neuropsychological test results. Based on the total CogEvo score, the cognitively normal and cognitive decline groups were identified by receiver operating characteristic curve analysis with a moderate predictive performance. The cognitive decline group was well identified using the orientation/spatial cognitive function factor.

**Conclusions:**

CogEvo is a valid and reliable screening tool for cognitive function evaluation. It proved useful in the early identification of cognitive decline in our study sample.

## Graphical abstract

CogEvo, a computerized cognitive function evaluation battery, consisted of two‐factor structures: orientation/spatial cognitive function and attention/executive function. Cognitive decline was identified using the total CogEvo score, orientation/spatial cognitive function factor score, and attention/executive function factor score with moderate predictive performance. Also, the prediction of more severe cognitive decline was identified using the total CogEvo score and orientation/spatial cognitive function factor score with moderate predictive performance.

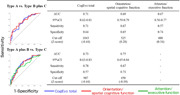

## INTRODUCTION

In Japan, the number of dementia patients is estimated to reach seven million by 2025.^1^ Alzheimer's disease (AD) is a progressive degenerative disease that irreversibly impairs the acquired intellectual function, resulting in severe cognitive dysfunction. Drugs that improve the symptoms have been used in such cases to suppress exacerbation and delay the progression of cognitive dysfunction. Recently, new amyloid disease modifiers that control progression have been developed.^2^, ^3^, ^4^ The first step in diagnosing cognitive dysfunction is to identify mild cognitive impairment (MCI) or the cognitively normal (CN) before the onset of AD, and then begin the treatment accordingly.^4^, ^5^, ^6^

So far, the Mini‐Mental State Examination (MMSE) and Montreal Cognitive Assessment (MoCA) have been known as the neuropsychological tests that detect cognitive decline and sensitively discriminate between MCI and AD.^7^, ^8^, ^9^, ^10^ These tests conducted are in‐person, and individuals respond to questions using paper and pencil. Evidently, these are expensive, as they demand skilled doctors and clinical psychologists as well as a longer duration of evaluation. In future in Japan, as the ratio of elderly people to medical personnel increases, it will not be easy to sufficiently screen elderly people for cognitive decline using conventional methods alone. Therefore, simple screening methods for cognitive assessment that can be administered to a higher number of such individuals are needed. Recently, batteries for computerized cognitive assessments using audiovisual tasks displayed on a monitor have been developed worldwide to replace the conventional neuropsychological tests, and these are being widely used in clinical trials.^11^, ^12^ In line with this trend, CogEvo (CogEvo Total Brain Care Co. Ltd) was developed in Japan, and to date, the association with conventional neuropsychological tests and the prediction accuracy of MCI and AD have been investigated.^13^, ^14^, ^15^ In CogEvo, five tasks (orientation, spatial cognition, attention, memory, and execution) presented on the touch panel monitor screen are performed, yielding computerized scores.^13^, ^14^ In addition to facilitating the accumulation and management of scores, this system comes with improved device usability. For instance, to ensure better and easier audibility for the elderly, it includes voice guidance. Additionally, it includes easily comprehensible instructions and color schemes based on color universal design.

This study examined CogEvo as a screening tool for cognitive decline involving 123 participants (ages 57–97). Five basic CogEvo tasks and two conventional, in‐person, questions‐based neuropsychological tests, namely, the MMSE and Trail Making Test (TMT), were performed and their correlations were analyzed, respectively. Moreover, the validity and reliability of CogEvo were examined using factor analysis, MacDonald's omega (*ω*) reliability coefficient, logistic regression analysis, and receiver operating characteristic (ROC) curve analysis. We also reported on the usefulness of CogEvo in identifying cognitive function decline at an early stage.

## METHODS

### Participants

This retrospective study was approved by the Institutional Review Board (IRB) of Satoh Neurosurgical Hospital, and the protocols used in this study were approved by the Committee of Human Subjects Protection of the Satoh Neurosurgical Hospital, Hiroshima, Japan (Code: IRB: 2022‐01, April. 20, 2022). All methods were performed in accordance with the relevant guidelines and regulations. Informed consent was obtained from all participants for using their clinical data. A total of 123 participants (mean age ± standard deviation [SD]: 80.35 ± 7.64 years; age range: 57–97 years; 77 women) underwent a screening test for cognitive function at our hospital; the study period spanned over 36 months, starting from April 2018 to March 2021. There were elementary and junior high school graduates (37; 30.08%), high school graduates (70; 56.91%), junior college and vocational school graduates (9; 7.32%) as well as university graduates and those with higher qualification (7; 5.69%). None of the participants had neurologic or psychiatric deficits due to overt cerebrovascular disease. They were all community‐dwelling elderly individuals who experienced subjective forgetfulness and cognitive decline.

### Neuropsychological tests

The following two neuropsychological tests were performed: short‐term MMSE (Japanese version)^16^; and TMT Part A (TMT‐A) with Part B (TMT‐B).^17^

### CogEvo test

CogEvo entailed five tasks: the “orientation” task evaluated orientation function (orientation to time); the “same shape” task evaluated visuospatial cognition (figure matching with mental rotation); the “follow the order” task evaluated visual attention function (sustained attention/divided attention/set‐shifting); the “flash light” task evaluated memory function (executive function, including working memory); and the “route 99” task evaluated executive function (strategy/planning/efficiency).^13^, ^14^ Each task was presented audiovisually on a touch‐panel monitor, and after the participants responded, the score was automatically calculated and displayed on the computer. The scoring is based on a unique evaluation system of correct answers and reaction time score.^13^ While one point is added for each correct response to a reaction in each task, the score for four tasks, excluding the flash light task, is calculated as follows: reaction time score = (standardized time limit − actual reaction time during task)/standardized time limit×100 points. The standardized time limit is set based on “average + 3 × SD” of preliminary survey data of 50,000 people. The maximum score for the orientation task result is 450 points, the same shape task is 600 points, the follow the order task is 455 points, the flash light task is 2100 points, and the route 99 task is 450 points.

### Statistical analysis

Means and standard deviations were calculated for the results of the two neuropsychological tests, the total score of CogEvo, and each score of the five tasks. Based on the result of the MMSE,^13^ for convenience, we classified cognitive decline into three groups: Type A group (MMSE >27), which included those for whom cognitive function decline was not suspected; Type C group (MMSE ≤23), which included those for whom cognitive decline was suspected; and Type B group (MMSE 24–26), which included those who were between Types A and C. Differences among the three groups were examined using the following tests; one‐way analysis of variance, Kruskal–Wallis test, and multiple comparisons with Bonferroni correction. Additionally, the total CogEvo score and each task score were corrected for variation owing to the difficulty and maximum score of the task using the standardized score (*Z*‐score) based on acquired data, followed by statistical processing from 1 to 4.

1. To verify the structural validity of CogEvo, an exploratory factor analysis (EFA) based on maximum‐likelihood factoring and Promax rotation was performed. The number of latent factors was estimated on the basis of the Scree plot, Kaiser–Gutmann standard, and reaching a cumulative contribution ratio of 50%. After determining the number of latent factors and their factor names, the factor structure was verified using confirmatory factor analysis (CFA). The *χ* ^2^ value, goodness‐of‐fit index (GFI), comparative fit index (CFI), and root‐mean‐square error of approximation (RMSEA) were used for the goodness of fit of the model. GFI and CFI were judged as good models when they were >0.9. RMSEA was very good when 0 ≤ RMSEA ≤ 0.05, and bad when 0.1 ≤ RMSEA. Various parameters of the analytical model were estimated using the maximum likelihood method.
2. The construct validity of CogEvo was examined using Pearson's or Spearman's partial correlation analysis adjusted for age, sex, and educational level. The relationship between the CogEvo‐related scores, the total MMSE score, and the time required for TMT were analyzed. The problem of multiplicity of tests that surfaced in the analyses was corrected by multiplying the calculated *p*‐vale by the number of tests (Bonferroni correction) to determine significance.
3. To verify the criterion‐related validity of CogEvo, multivariate logistic regression analyses were conducted with the presence or absence of suspicion of cognitive decline (0: Type A vs. 1: Type B plus C) or more severe cognitive decline (0: Type A plus B vs. 1: Type C) as an outcome variable and variables simultaneously entered from age, gender, educational level, and total CogEvo score/factor scores as predictor variables. To confirm the usefulness of CogEvo, ROC curve analysis was performed. The area under the curve (AUC), 95% confidence interval (95% CI), sensitivity, specificity, and cut‐off value (the point closest to the upper left corner) were calculated.
4. Internal consistency was confirmed by McDonald's *ω* coefficient, which is considered adequate with a value of 0.6 or higher.

IBM SPSS Statistics and Amos Version 25 (SPSS), EZR Version 1.11 (https://www.jichi.ac.jp/saitama-sct/SaitamaHP.files/statmed.html), and JASP Version 0.16 (https://jasp-stats.org/) were used for statistical analysis. The significance level (α) for all statistical tests was set at 0.05.

## RESULTS

### Relationship between CogEvo and neuropsychological tests

Table 1 shows the demographic data, neuropsychological test scores, and CogEvo scores. There were no significant differences in age, sex, or educational level among the three groups of MMSE (Type A, Type B, and Type C). The total MMSE score was 25.16 ± 4.23, and the scores decreased significantly in the order of Type A (28.50 ± 1.20), Type B (25.07 ± 0.72), and Type C (19.51 ± 2.95) (*p* < 0.05) groups. In the TMT‐A, the Type C group (244.91 ± 157.00) performed significantly worse than the Type B (173.00 ± 78.96) and Type A groups (130.95 ± 46.98) (*p* < 0.05). In TMT‐B, the Type C group (201.69 ± 87.24) showed a significant decrease compared to the Type A group (194.80 ± 98.82) (*p* < 0.05) and marginally significant decrease compared to the Type B group (283.14 ± 109.07) (*p* < 0.10). Missing values were observed in the TMT‐A (*n* = 1) and TMT‐B (*n* = 34) because participants could not fully understand the test content, or could not meet the time limit at all, or refused to complete it.

**Table 1:** MMSE group comparison of demographic data, neuropsychological test, and CogEvo

	Total	Type A group	Type B group	Type C group	Group comparison
		MMSE ≥27	MMSE 24–26	MMSE ≤23	
Sample	123	60	28	35	‐
Age (years)	80.35 ± 7.64	79.27 ± 7.75	81.61 ± 7.51	81.20 ± 7.52	*F*_2, 120_ = 1.20, n.s.
Number of females	77 (62.60%)	36 (60.00%)	17 (60.71%)	24 (68.57%)	*χ* ^2^_2_ = 0.69, n.s.
Education level (Range: 1–4)	1.80	1.85	1.73	1.80	H_2_ = 0.78, n.s.
MMSE total (/30)	25.16 ± 4.23	28.50 ± 1.20	25.07 ± 0.72	19.51 ± 2.95	*F*_2, 120_ = 270.75*
					A > B*, B > C*, A > C*
TMT‐A time (s)	172.36 ± 107.33 (*n* = 122)	130.95 ± 46.98 (*n* = 60)	173.00 ± 78.96 (*n* = 28)	244.91 ± 157.00 (*n* = 34)	*F*_2, 119_ = 15.08*
					B > C*, A > C*
TMT‐B time (s)	217.64 ± 105.78 (*n* = 89)	194.80 ± 98.82 (*n* = 54)	283.14 ± 109.07 (*n* = 22)	201.69 ± 87.24 (*n* = 13)	*F*_2, 86_ = 6.30*
					A > B^+^, A > C*
CogEvo total	1021.82 ± 264.13 (0.00 ± 1.00)	1139.45 ± 222.68 (0.45 ± 0.84)	961.36 ± 247.27 (−0.23 ± 0.94)	868.54 ± 253.22 (−0.58 ± 0.96)	*F*_2, 120_ = 15.58*
					A > B*, A > C*
Orientation	234.37 ± 99.37 (0.00 ± 1.00)	279.43 ± 79.04 (0.45 ± 0.80)	228.18 ± 81.88 (−0.06 ± 0.82)	162.09 ± 101.35 (−0.73 ± 1.02)	*F*_2, 120_ = 20.41*
					A > B*, B > C*, A > C*
Same shape	246.56 ± 93.10 (0.00 ± 1.00)	273.37 ± 89.57 (0.29 ± 0.96)	223.11 ± 81.44 (−0.25 ± 0.87)	219.37 ± 97.13 (−0.29 ± 1.04)	*F*_2, 120_ = 5.20*
					A > B*, A > C*
Flash light	174.20 ± 119.14 (0.00 ± 1.00)	196.07 ± 108.46 (0.18 ± 0.91)	151.18 ± 102.31 (−0.19 ± 0.86)	155.11 ± 143.37 (−0.16 ± 1.20)	n.s.
Follow the order	195.61 ± 54.64 (0.00 ± 1.00)	215.73 ± 58.83 (0.37 ± 1.08)	184.07 ± 45.43 (−0.21 ± 0.83)	170.34 ± 39.83 (−0.46 ± 0.73)	*F*_2, 120_ = 9.63*
					A > B*, A > C*
Route 99	171.08 ± 51.28 (0.00 ± 1.00)	174.85 ± 50.22 (0.07 ± 0.98)	174.82 ± 49.70 (0.07 ± 0.97)	161.63 ± 54.50 (−0.18 ± 1.06)	n.s.

*Note*: The education level in the table shows the median; the other values show the mean ± standard deviation. TMT entails missing values, and the number of samples is shown in parentheses. CogEvo totals and subtasks are also shown in parentheses for *Z*‐score.

Abbreviations: MMSE, Mini‐Mental State Examination; TMT, Trail Making Test.

*p < 0.05; ^+^ *p* < 0.10.

The total CogEvo score (1021.82 ± 264.13) was significantly lower in the Type B (961.36 ± 247.27) and Type C (858.54 ± 253.32) groups than in the Type A group (1139.45 ± 222.68) (*p* < 0.05). No significant differences were observed between the Type B and Type C groups in this regard. The scores of the five CogEvo tasks significantly decreased in the order of Type A, Type B, and Type C groups in the orientation task (*p* < 0.05). In the same shape and follow the order tasks, a significant decrease was observed in the Type B and Type C groups compared to the Type A group (*p* < 0.05) but not between the Type B and Type C groups. In the flash light and route 99 tasks, no significant difference was found among the three groups.

### Verification of structural validity and reliability of CogEvo

**Figure 1:**
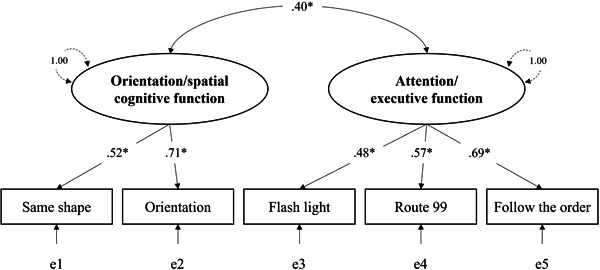
Result of confirmatory factor analysis in a two‐factor correlation model. Note: the numerical values in the figure are standardization coefficients; all were significant paths. *e*, error variable, *p < 0.05.

EFA with five items of CogEvo revealed two latent factors, including “orientation/spatial cognitive function,” composed of the orientation and same shape tasks, and “attention/executive function,” composed of the follow the order, flash light, and route 99 tasks (Table 2). The two latent variables were named after the functions evaluated in CogEvo's five tasks. Both factors were significantly correlated (*r* = 0.26, *p* < 0.05). The results of CFA using the two‐factor correlation model showed statistically acceptable levels by the goodness of fit of the model, with *χ* ^2^ (4) = 5.74 (*p* = 0.22), GFI = 0.98, CFI = 0.97, RMSEA = 0.06 (Figure 1). MacDonald's *ω* reliability coefficient was 0.60 for the two tasks on orientation/spatial cognitive function factor, 0.62 for the three tasks on attention/executive function factor, and 0.60 for all tasks (Table 2). Orientation/spatial cognitive function factor score was significantly lower in the Type B (451.29 ± 130.54) and Type C (381.46 ± 144.20) groups than in the Type A group (552.80 ± 145.59) (*p* < 0.05), while attention/executive function factor score was significantly lower in the Type C group (487.09 ± 183.91) compared to the Type A group (586.65 ± 165.26) (*p* < 0.05).

**Table 2:** Results of EFA (factor pattern after Promax rotation), interfactor correlations, and MMSE group comparison of factor score

	Factor loading		Communality
Total (*ω* = 0.60)	Factor 1	Factor 2	
First factor: *ω* = 0.60			
Same shape	**0.841**	−0.075	0.678
Orientation	**0.425**	0.144	0.235
Second factor: *ω* = 0.62			
Route 99	−0.116	**0.691**	0.449
Follow the order	0.084	**0.619**	0.417
Flash light	0.204	**0.403**	0.247
Inter‐factor correlations	Factor 1	Factor 2	
Factor 1	1.000	–	
Factor 2	**0.263** *	1.000	

Factor	Type A group:	Type B group:	Type C group:	Group comparison
	MMSE ≥27	MMSE 24–26	MMSE ≤23	
Factor 1: Orientation/spatial cognitive function	552.80 ± 145.59	451.29 ± 130.54	381.46 ± 144.20	*F*_2,120_ = 16.90*
				A > B*, A > C*
Factor 2: Attention/executive function	586.65 ± 165.26	510.07 ± 148.07	487.09 ± 183.91	*F*_2,120_ = 4.54*
				A > C*

*Note*: *ω*, McDonald's omega coefficient. Bold values used to highlight the factor loadings.

Abbreviations: EFA, exploratory factor analysis; MMSE, Mini‐Mental State Examination.

*p < 0.05.

### Verification of the construct validity of CogEvo

Partial correlation analysis was performed to verify the construct validity of CogEvo (Table 3). The orientation/spatial cognitive function factor and the total CogEvo score showed a significant correlation with total MMSE score, and TMT‐A and TMT‐B performance (*p* < 0.05). The attention/executive function factor score was significantly associated with the total MMSE score and TMT‐A performance (*p* < 0.05).

**Table 3:** Partial correlation analysis results between CogEvo‐related scores and neuropsychological test performance

	CogEvo total	Orientation/spatial cognitive function	Attention/executive function
MMSE total^a^	**0.45** *	**0.46** *	**0.28** *
TMT‐A time (sec)	**−0.46** *	**−0.37** *	**−0.36** *
TMT‐B time (sec)	**−0.40** *	**−0.38** *	−0.21

*Note*: The numbers in the table show the correlation coefficient adjusted for age, sex, and education level. Bold text emphasize statistically significant values.

^a^ Spearman's rank correlation analysis.

Abbreviations: MMSE, Mini‐Mental State Examination; TMT, Trail Making Test.

* *p* < 0.05.

### Verification of CogEvo criterion‐related validity: Predictive analysis of cognitive decline with CogEvo

Predictions of cognitive decline (discriminating the Type A group from the Type B group plus Type C group) and the presence or absence of more severe cognitive decline (discriminating Type A group plus Type B group from Type C group) were examined using multivariate logistic regression analysis and ROC curve analysis (Table 4). Cognitive decline was significant with the total CogEvo score (odds ratio [OR] 2.21, CI 1.37–3.56, *p* < 0.05) and orientation/spatial cognitive function factor score (OR 1.78, CI 1.14–2.78, *p* < 0.05), while marginally significant with attention/executive function factor score (OR 1.52, CI 0.96–2.40, *p* < 0.1). Regarding the presence or absence of more severe cognitive decline, the total CogEvo (OR 2.81, CI 1.66–4.76, *p* < 0.05) and orientation/spatial cognitive function factor score (OR 2.64, CI 1.59–4.39, *p* < 0.05) were statistically significant. However, the attention/executive function factor score was not significantly associated with more severe cognitive decline.

**Table 4:** Results of multiple logistic regression analysis

Explanatory variable	Presence of cognitive decline （Type A vs. Type B + Type C）		Presence of more severe cognitive decline （Type A + Type B vs. Type C）	
	OR	95% CI	OR	95% CI
Model 1: CogEvo one factor				
Age	0.98	0.92–1.04	1.04	0.97–1.11
Sex	0.89	0.39–2.07	0.66	0.25–1.73
Education level	1.00	0.58–1.72	1.25	0.70–2.26
CogEvo total: *Z*‐score	**2.21** *	**1.37–3.56**	**2.81** *	**1.66–4.76**
Model 2: CogEvo two factors				
Age	0.97	0.91–1.03	1.03	0.96–1.10
Sex	0.93	0.40–2.17	0.79	0.29–2.12
Education level	1.00	0.58–1.72	1.27	0.70–2.29
Orientation/spatial cognitive function: *Z*‐score	**1.78** *	**1.14–2.78**	**2.64** *	**1.59–.39**
Attention/executive function: *Z*‐score	1.52^+^	0.96–2.40	1.39	0.86–2.26

*Note*: Type A: MMSE ≥27, Type B: MMSE 24–26, Type C: MMSE ≤23. Bold text emphasize statistically significant values.

Abbreviations: CI, confidence interval; MMSE, Mini‐Mental State Examination; OR, odds ratio.

*p < 0.05; ^+^ *p* < 0.10.

**Figure 2:**
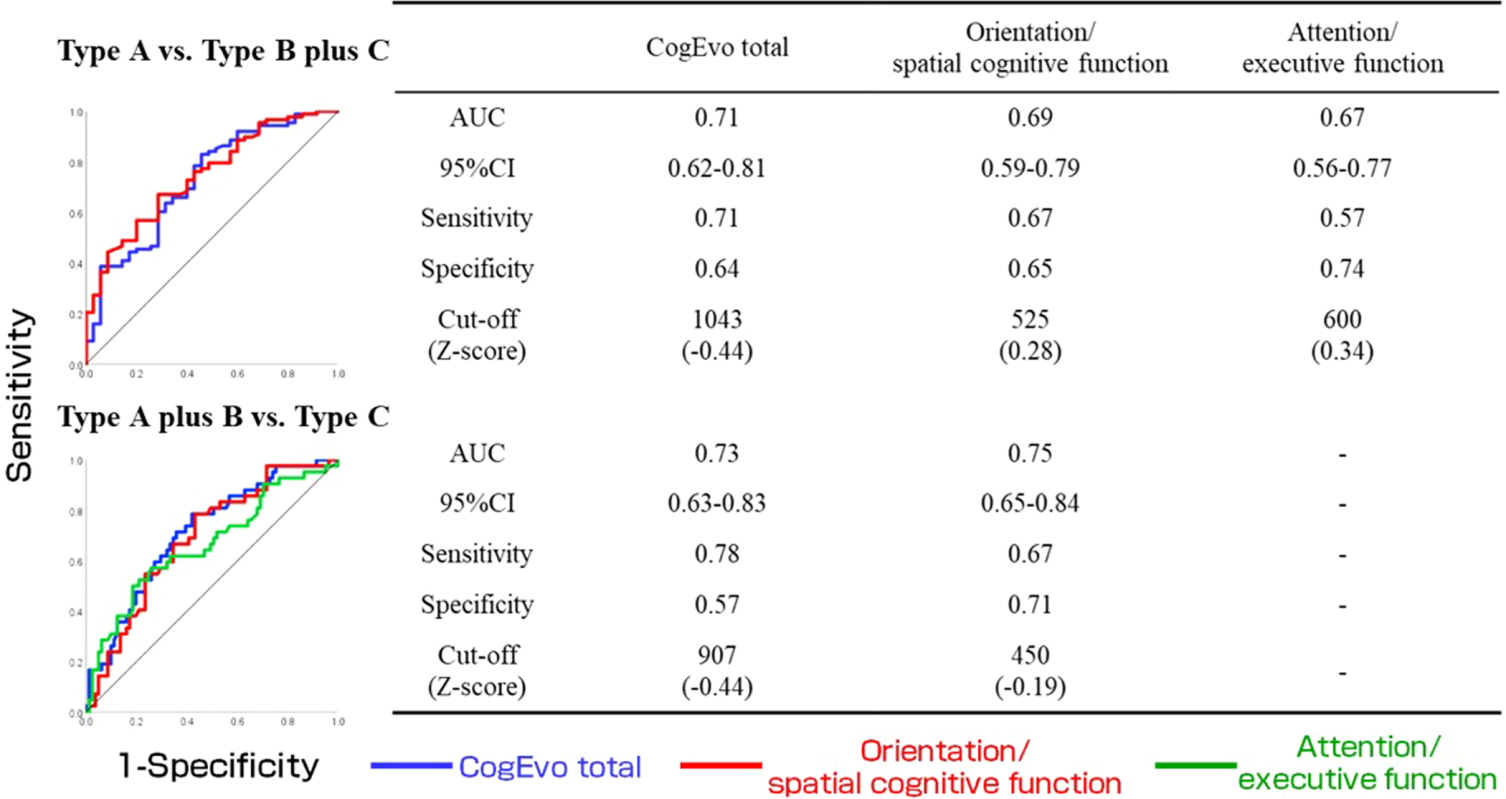
Results of receiver operating characteristic (ROC) analysis. Type A: Mini‐Mental State Examination (MMSE) ≥27, Type B: MMSE 24–26, Type C: MMSE ≤23. Abbreviations: AUC, area under the curve; CI, confidence interval; CogEvo, cognitive function evaluation.

Predictability was evaluated by ROC curve analysis using the predictor variables of CogEvo associated with cognitive decline and more severe cognitive decline obtained by multivariate logistic regression analysis (Figure 2). Cognitive decline was identified using the total CogEvo score (AUC = 0.71, sensitivity 71%, specificity 64%, cut‐off value −0.44/raw score 1043 points), orientation/spatial cognitive function factor score (AUC = 0.69, sensitivity 67%, specificity 65%, cut‐off value 0.28/raw score 525 points), and attention/executive function factor score (AUC = 0.67, sensitivity 57%, specificity 74%, cut‐off value 0.34/raw score 600 points), indicating moderate predictive performance. Prediction of more severe cognitive decline was identified using the total CogEvo score (AUC = 0.73, sensitivity 78%, specificity 57%, cut‐off value −0.44/raw score 907 points) and orientation/spatial cognitive function factor score (AUC = 0.75, sensitivity 67%, specificity 71%, cut‐off value −0.19/raw score 450 points), indicating moderate predictive performance.

## DISCUSSION

CogEvo is a computerized cognitive function evaluation battery newly developed in Japan for screening and training of cognitive functions.^13^, ^14^, ^15^ In CogEvo, audio‐visual tasks, including orientation (orientation of time), same shape (visuospatial cognition), follow the order (visual attention), flash light (working memory), and route 99 (executive function), are performed. The participants face a touch panel monitor screen and are presented with audiovisual tasks. The grade of each task is quantified and scored using a computer. Unlike conventional face‐to‐face question‐type neuropsychological tests, such as the MMSE and MoCA, CogEvo does not necessarily require a specialized doctor or clinical psychologist.^6^, ^13^, ^14^ By performing CogEvo sequentially, the evaluation at the time of the test and the changes in cognitive function over time can be obtained.^15^ The score data are recorded automatically and accumulate over time.

Ichii et al.^13^ reported that CogEvo and MMSE scores showed a significant relationship in 272 participants. ROC curve analysis in our study showed that the total CogEvo score could discriminate cognitive decline, such as levels of MCI and dementia. Similarly, Takechi and Yoshino^14^ reported that the cognitive decline group (MCI and AD) could be discriminated from the CN group with high accuracy based on the total CogEvo score. Since the MMSE is evaluated using a deduction formula with a maximum of 30 points, a ceiling effect is likely to occur.^6^, ^8^, ^9^, ^10^ The MoCA, which is superior to the MMSE in detecting MCI, including various cognitive domains (i.e., executive functions like TMT‐B, phonemic fluency, and abstraction),^8^ also moderates the ceiling effect somewhat, but it is considered to be influenced by educational bias.^9^, ^18^ Moreover, tests that evaluate cognitive domains that are not included in the MMSE, such as TMT, which were performed supplementarily in this study, do so by reaction time and correct answers, as in CogEvo. However, they are likely to produce missing values due to the time limits, as also occurred in this study. The detection capacity of slight cognitive decline in the preclinical period may decrease if only conventional, in‐person, questions‐based neuropsychological tests are relied upon.^6^, ^9^, ^12^, ^13^ On the contrary, the ceiling effect and missing values are unlikely to occur with CogEvo because the scores are computerized mainly based on the accuracy and reaction time to tasks. Thus, CogEvo can be an effective tool for assessing age‐specific risk of cognitive decline in community‐dwelling elderly people as well as, or even better than, the conventional in‐person, question‐based neuropsychological tests.

### Validity and reliability of CogEvo

New information was provided in this study in the form of the two‐factor structure revealed by the factor analysis, including the orientation/spatial cognitive function and attention/executive function factors in CogEvo. Construct validity was confirmed by partial correlation analysis, in which the total CogEvo score and orientation/spatial cognitive function and attention/executive function factor scores were related to the total MMSE score and TMT scores. Additionally, criterion‐related validity was confirmed by ROC curve with moderate predictive performance analyses in which the CN and cognitive decline groups were discriminated. Particularly, the cognitive decline group was well identified by the orientation/spatial cognitive function factor. The reliability of CogEvo was guaranteed with a MacDonald's omega coefficient of approximately 0.60. Based on these results, it is possible to evaluate cognitive decline in community‐dwelling elderly people using the total CogEvo score, orientation/spatial cognitive function, and attention/executive function factor scores.

### Prediction of cognitive decline with CogEvo

Prediction of the decline in cognitive function (discrimination between the Type A group and the Type B group plus Type C group) and the presence or absence of more severe cognitive decline (Type C group and Type A group plus Type B group) using the total CogEvo, orientation/spatial cognitive function factor, and attention/executive function factor scores were examined using multivariate logistic regression and ROC curve analysis. The area under the ROC curve (AUC) for predicting the presence or absence of cognitive decline yielded 0.71 (cut‐off value/*Z*‐score: 1043 points/−0.44) for the total CogEvo score, 0.69 (cut‐off value/*Z*‐score: 525 points/0.28) for the orientation/spatial cognitive function factor score, and 0.67 (cut‐off value/*Z*‐score: 600 points/0.34) for the attention/executive function factor score. Thus, moderate predictive performance was identifiable using CogEvo among community‐dwelling elderly people. Takechi and Yoshino^14^ reported similar results, that is, the MCI and AD groups and the CN group could be distinguished by the total score of CogEvo with moderate predictive performance (AUC 0.83). The AUC for predicting the presence or absence of more severe cognitive decline was 0.73 (cut‐off value/*Z*‐score: 907 points/−0.44) for the total CogEvo score and 0.75 (cut‐off value/*Z*‐score: 450 points/−0.19) for the orientation/spatial cognitive function factor score. Relatively more severe cognitive decline could be predicted by the total CogEvo score (cut‐off value: 907 points) and orientation/spatial cognitive function factor score (cut‐off value: 450 points). A decrease in the orientation/spatial cognitive function factor score was considered a useful discriminant index in evaluating the orientation^19^, ^20^ and spatial cognitive function^21^ of the atrophic temporal and parietal lobe in typical AD,^22^ while the attention/executive function factor scores alone did not have the ability to predict more severe cognitive decline, like dementia. However, in the case of the frontal variant of AD,^22^ the attention/executive function factor might play a role in evaluating the frontal cognitive function.^23^

### Limitation and future prospects

In this study, based on the overall CogEvo score, it was possible to identify the cognitive decline group (Type A vs. Type B plus Type C) and the more severe cognitive decline group (Type A plus Type B vs. Type C) was classified based on the total MMSE cut‐off. Since the study participants did not clinically establish a diagnosis of MCI or dementia, the validity and predictive ability of the two‐factor structure of orientation/spatial cognitive function and attention/executive function need further investigation. It is important to evaluate the validity and reliability of CogEvo in the long term by repeating it and tracking temporal changes in cognitive decline. The use of computerized evaluation tools is expected to cause tension and anxiety in elderly people with cognitive decline due to unknown and unfamiliar elements. However, CogEvo has great merit because it does not necessarily require skilled doctors and clinical psychologists, and the evaluation time can be reduced.^6^, ^15^ Moreover, CogEvo is expected to continue to be used in clinical research, as it can ensure quantitativeness, objectivity, and reproducibility. It also enables cognitive training in a game‐like format.^15^

## CONCLUSION

The validity and reliability of the computerized cognitive function evaluation battery, CogEvo, was confirmed as a cognitive decline screening tool. In CogEvo, the two‐factor structure consisting of orientation/spatial cognitive function and attention/executive function was a novel find. CogEvo can be considered a useful cognitive evaluation battery for early identification of mild/moderate cognitive decline.

## AUTHOR CONTRIBUTIONS

Yoichi Sawada conducted statistical analyses and prepared Tables 1, 2, 3, 4 and Figures 1, 2. Toru Satoh assisted in the preparation of Table 1 and Figures 1, 2. Hideaki Saba, Hiroshi Kitamoto, Yoshiki Kato, Yoshiko Shiozuka, Tomoko Kuwada, Megumi Sasaki, Kana Murakami, and Yudai Abe discussed the results with Yoichi Sawada, Toru Satoh, and Kaori Harano. Yoichi Sawada and Toru Satoh wrote the manuscript. Yoichi Sawada and Toru Satoh designed and organized the study. All authors contributed to the analysis and interpretation of data. All authors critically reviewed the manuscript.

## CONFLICT OF INTEREST

The authors declare no conflict of interest.

## ETHICS APPROVAL STATEMENT

This retrospective study was approved by the Institutional Review Board (IRB) of Satoh Neurosurgical Hospital, and the protocols used in the study were approved by the Committee of Human Subjects Protection of the Satoh Neurosurgical Hospital, Hiroshima, Japan (Code: IRB: 2022‐01, April. 20, 2022). All methods were performed in accordance with the relevant guidelines and regulations.

## PATIENT CONSENT STATEMENT

Informed consent has been obtained from all participants for using their clinical data.

## CLINICAL TRIAL REGISTRATION

N/A

## Data Availability

The datasets generated and/or analyzed during the current study can be obtained from the corresponding author on reasonable request.
